# Differential ROS Accumulation, Antioxidant Defense, and Physiological Adaptation in Two *Rosa rugosa* Cultivars Under Sustained Salt Stress

**DOI:** 10.3390/antiox15091218

**Published:** 2026-09-21

**Authors:** Sawaira Ashraf, Muhammad Zahid Ashraf, Zhonghua Bian, Humera Ashraf, Waqar Ahmed, Baohe Miao, Xinxin Zhao

**Affiliations:** 1 Institute of Urban Agriculture, Chinese Academy of Agricultural Sciences, Chengdu 610213, China; sawairaashraf717@gmail.com (S.A.); bianzhonghua@caas.cn (Z.B.); 2 State Key Laboratory for Conservation and Utilization of Subtropical Agro-Bioresources, South China Agricultural University, Guangzhou 510642, China; ashrafzahid848@gmail.com; 3 Crop Research Institute, Guangdong Academy of Agricultural Sciences, Guangzhou 510640, China; humeraa1990@gmail.com; 4 National Institute of Food Science and Technology, University of Agriculture, Faisalabad 38000, Pakistan; 729waqar@gmail.com

**Keywords:** antioxidant enzymes, lipid peroxidation, osmolyte and ROS accumulation, *Rosa rugosa*, salt stress, salt tolerance

## Abstract

Salt stress is a major abiotic constraint that limits plant growth by inducing oxidative stress, disrupting photosynthetic processes, and damaging cellular membranes. Although *Rosa rugosa* is considered relatively tolerant to adverse environments, differences in antioxidant defense and physiological responses among its cultivars under sustained salt stress remain insufficiently understood. Comparative evaluation of these responses is important for identifying salt-tolerant germplasm and understanding stress-adaptation mechanisms. In this study, two *R. rugosa* cultivars, Siji and Fenghua, were subjected to irrigation treatments with solutions containing 0, 50, 100, or 150 mM NaCl for 28 days under controlled conditions. Morphological traits, salt injury, photosynthetic pigments, osmolytes, ROS accumulation, antioxidant enzyme activities, and lipid peroxidation were measured to assess cultivar-specific differences in salt-stress responses. Salt stress caused concentration-dependent growth inhibition, increased salt injury, and reduced chlorophyll content, while oxidative-stress responses differed between the two cultivars, with stronger adverse effects observed in Fenghua. Siji maintained better growth performance, higher fresh and dry mass of sampled leaves, greater chlorophyll retention, better maintenance of several osmolytes, and stronger antioxidant enzyme activities under the higher NaCl treatments. In contrast, Fenghua showed greater ROS accumulation and higher MDA under the higher NaCl treatments, which are consistent with greater oxidative stress and membrane lipid peroxidation. Exploratory correlation and principal component analyses further showed that antioxidant defense, osmotic adjustment, chlorophyll retention, and reduced oxidative damage were closely associated with salt tolerance in Siji. These results indicate that greater antioxidant capacity, osmotic adjustment, and membrane protection were associated with the superior salt-stress performance of ‘Siji’ compared with ‘Fenghua’. The study identifies Siji as a promising *R. rugosa* candidate for further salt-tolerance evaluation and provides useful physiological and biochemical indicators for salt-tolerance screening in ornamental rose breeding.

## 1. Introduction

Soil salinity is one of the major abiotic stresses limiting plant growth and productivity worldwide [1]. Salinity affects more than 20% of irrigated agricultural land, and its extent is expected to increase because of climate change, inappropriate irrigation practices, and soil degradation [2]. High salt concentrations in soil cause osmotic stress and ion toxicity, thereby reducing water uptake and disrupting nutrient balance and cellular metabolism [3]. These effects ultimately result in growth inhibition and reduced plant performance [4]. One of the major consequences of salinity stress is excessive production of reactive oxygen species (ROS) [5]. Among ROS, hydrogen peroxide (H_2_O_2_) and superoxide anion (O_2_^−^) are particularly important indicators of salt-induced oxidative stress [6]. Superoxide anion is rapidly generated under stress conditions and can be transformed by superoxide dismutase into H_2_O_2_, whereas H_2_O_2_ is subsequently scavenged by catalase, peroxidase, and ascorbate peroxidase [7,8]. Though H_2_O_2_ can function as a signaling molecule at controlled levels, excessive accumulation of O_2_^−^ and H_2_O_2_ interrupts cellular redox homeostasis and promotes oxidative injury [9]. Therefore, direct measurement of O_2_^−^ and H_2_O_2_, together with antioxidant enzyme activities and MDA content, provides a more complete assessment of oxidative stress intensity and ROS-scavenging efficiency under salinity. The accumulation of ROS grounds oxidative damage to cellular components such as lipids, proteins, and nucleic acids, thereby impairing membrane integrity and cellular functions [5,10]. To minimize oxidative damage, plants trigger sophisticated antioxidant defense systems which include enzymatic antioxidants such as superoxide dismutase (SOD), peroxidase (POD), catalase (CAT) and ascorbate peroxidase (APX) [11,12]. These enzymes play important roles in ROS scavenging and the maintenance of cellular redox homeostasis under stress. Besides the antioxidant defense mechanisms, plants employ several physiological strategies to tolerate salinity stress. Osmotic adjustment is an important adaptive response that is associated with accretion of compatible solutes such as proline (PRO), soluble sugars (SS) and soluble proteins (SP) [13]. These osmolytes contribute to osmotic balance, alleviate proteins and membranes, and defend cellular structures under stress conditions. Furthermore, salinity-induced oxidative stress often leads to lipid peroxidation of cellular membranes, which can be assessed by measuring malondialdehyde (MDA), a widely used indicator of membrane damage [6]. Chlorophyll content is another important physiological parameter affected by salinity, as salt stress can disrupt chloroplast structure and reduce photosynthetic efficiency [14].

*R. rugosa* is an important ornamental and ecological species widely cultivated for its esthetic value, fragrance, and adaptability to diverse environmental conditions [15]. It is commonly used in landscaping, coastal protection, and ecological restoration due to its tolerance to harsh environments [16,17]. However, increasing soil salinity in many regions poses a significant challenge to the growth and ornamental quality of *R. rugosa* [18,19]. Although *R. rugosa* has been investigated for its responses to salinity, cultivar-level differences in coordinated physiological and biochemical responses remain insufficiently resolved, particularly under prolonged NaCl exposure. Previous studies have mainly focused on general stress responses or individual plant materials, whereas direct comparisons between *R. rugosa* cultivars are still limited [18].

Therefore, the present study compared *Rosa rugosa* ‘Siji’ and *Rosa rugosa* ‘Fenghua’ to determine whether differences in growth performance under sustained NaCl treatment were associated with variation in ROS accumulation, antioxidant enzyme activities, osmolyte levels, chlorophyll retention, and lipid peroxidation. Growth traits, leaf area, leaf fresh weight, and leaf dry weight, salt injury, photosynthetic pigments, osmolytes, ROS accumulation, antioxidant enzyme activities, and MDA content were evaluated, and the resulting data were further examined using correlation and principal component analyses to identify response patterns associated with differential salt tolerance.

## 2. Materials and Methods

### 2.1. Plant Materials and Experimental Design

One-year-old clonally propagated, own-rooted plants of *R. rugosa* cultivars ‘Siji’ and ‘Fenghua’ were obtained from Jinan Tianhui Meigui Biological Technology Co., Ltd., Jinan, Shandong, China. The plants were supplied under their respective commercial cultivar names. Uniform, healthy plants were selected and cultivated under controlled conditions at the Institute of Urban Agriculture, Chinese Academy of Agricultural Sciences, Chengdu, Sichuan, China (30.4133° N, 104.1215° E). The plants were grown individually in plastic pots (16 cm in diameter), each containing approximately 2.4 L of a peat-based substrate composed of sphagnum peat supplemented with perlite, lime, and starter fertilizer. The pots had multiple drainage openings at the base and were placed on individual saucers. The experiment followed a completely randomized design (CRD) comprising two *R. rugosa* cultivars, four NaCl concentrations, and three biological replicates per treatment, resulting in 24 experimental units. Each experimental unit consisted of one independent pot containing one plant. Plants were randomly assigned to treatments and the positions of the pots were periodically rearranged to minimize positional effects. Measurements obtained from repeated assay wells were averaged to produce one value for each biological replicate. Plants were kept at 22 °C, 50% relative humidity, and a 16 h light/8 h dark photoperiod throughout the experimental period.

### 2.2. Salt Stress Treatment

After plant establishment, NaCl irrigation treatments were imposed gradually during the first week to minimize abrupt osmotic shock. The final NaCl concentrations of the irrigation solutions were 0 mM (control), 50 mM, 100 mM, and 150 mM. On day 1, plants assigned to the final 50, 100, and 150 mM NaCl treatments received irrigation solutions containing 25, 50, and 75 mM NaCl, respectively. The corresponding concentrations were increased to 50, 75, and 100 mM on day 3 and to 50, 100, and 125 mM on day 5. The final irrigation-solution concentrations of 50, 100, and 150 mM NaCl were reached on day 7 and were subsequently maintained for the remainder of the 28-day treatment period. Illustrative whole-plant phenotypes of ‘Siji’ and ‘Fenghua’ under each NaCl treatment are provided in Figure S1. Each pot received 400 mL of the respective NaCl solution every two days for 28 days. Control plants received an equal volume of tap water on the same schedule. On day 28, leaves from the second node were collected, immediately frozen in liquid nitrogen, and stored at −80 °C for further physiological and biochemical analyses.

### 2.3. Morphological Measurements

Morphological parameters were recorded repeatedly on the same individual plants, at 7-day time intervals (0, 7, 14, 21, and 28 days respectively, after salt treatment). The growth parameters considered were plant height, number of leaves, number of branches and stem diameter. Plant height was assessed from the stem base to the apex with a ruler. The number of leaves and branches per plant were counted manually. Stem diameter was measured with a digital caliper. Leaf Area (LA), leaf fresh weight (LFW) and leaf dry weight (LDW) were determined on the 28th day of the experiment due to the destructive nature of sampling. One fully expanded leaf of comparable developmental stage was collected from each plant (one leaf per biological replicate; *n* = 3 per cultivar × NaCl treatment combination) and used for all three measurements. Leaf area was measured using a leaf area meter (LI-3100C Area Meter, LI-COR Biosciences, Lincoln, NE, USA). The same sampled leaves were weighed immediately after harvest to determine LFW and were subsequently oven-dried at 70 °C to constant weight to determine LDW.

### 2.4. Salt Damage Score Evaluation

Salt injury was evaluated repeatedly on the same individual plants at days 0, 7, 14, 21, and 28 of the treatment periods. The salt injury scoring system was adapted from a previously published method [18]. Injury severity was assessed based on leaf chlorosis, wilting, curling, leaf abscission, and plant mortality. Each plant was assigned a salt injury index (SII) using an eight-level ordinal scale ranging from 0 to 7: 0, no visible injury; 1, approximately 10% of leaves showing chlorosis; 2, approximately 20% of leaves showing chlorosis; 3, 30–40% of leaves showing chlorosis with slight wilting or curling; 4, 50–60% of leaves showing chlorosis with evident wilting or curling and occasional leaf abscission; 5, 70–80% of leaves showing severe damage with substantial leaf abscission; 6, approximately 90% of leaves showing severe damage with extensive leaf abscission; and 7, plant death. All visual assessments were performed by the same researcher throughout the experiment to maintain scoring consistency. The proportion of affected leaves was estimated visually as the number of symptomatic leaves relative to the total number of leaves on each plant.

Physiological and biochemical assays were performed by means of commercial assay kits acquired from Geruisi Biotechnology Co., Ltd. (Suzhou, China); www.geruisi-bio.com. Fresh leaf tissue was used for all assays, and extraction and reaction procedures were performed according to the corresponding kit protocols, as described below. Microplate-based absorbance measurements were performed using an Epoch 2 microplate reader (BioTek Instruments, Inc., Winooski, VT, USA). Each biological replicate was analyzed in duplicate, and the two technical measurements were averaged to obtain one value for each biological replicate. Because the biochemical assays were performed on freshly harvested leaf tissue and quantified according to the assay-specific protocols results were expressed on a fresh-weight basis.

#### 2.4.1. Photosynthetic Pigments

##### Determination of Chlorophyll Content

Chlorophyll a (Chl a), chlorophyll b (Chl b), and total chlorophyll (Chl a + b) contents were determined using a commercial chlorophyll assay kit (G0601W; Geruisi Biotechnology Co., Ltd., Suzhou, China) based on pigment extraction [20]. Approximately 0.10 g of fresh leaf tissue, with the main vein removed, was homogenized under low-light conditions with 1 mL of extraction buffer consisting of ethanol and distilled water (95:5, *v*/*v*) and approximately 50 mg of the kit reagent. The homogenate and subsequent rinses were transferred to a glass tube and brought to a final extraction volume of 10 mL with extraction buffer. Samples were extracted in darkness for 3 h until the tissue residue was completely decolorized. No centrifugation was required for the resulting clear extract. For measurement, 200 μL of extract was transferred to a 96-well plate, with extraction buffer used as the blank, and absorbance was measured at 665 and 649 nm. Chlorophyll contents were calculated as follows:

$$\mathrm{Chlorophyll}\;a\;\mathrm{content}\;(\mathrm{mg}\;g^{-1}\;\mathrm{FW})=\frac{C_{a}\times V\times D}{1000\times W}$$

$$\mathrm{Chlorophyll}\;b\;\mathrm{content}\;(\mathrm{mg}\;g^{-1}\;\mathrm{FW})=\frac{C_{b}\times V\times D}{1000\times W}$$

$$\mathrm{Total}\;\mathrm{Chlorophyll}\;a+b\;\mathrm{content}\;(\mathrm{mg}\;g^{-1}\;\mathrm{FW})=\frac{C_{\left(a+b\right)}\times V\times D}{1000\times W}$$

where C_a_ = 13.95 × ΔA_665_ − 6.88 × ΔA_649_ (mg/L), C_b_ = 24.96 × ΔA_649_ − 7.32 × ΔA_665_ (mg/L), C_(a + b)_ = 6.63 × ΔA_665_ + 18.08 × ΔA_649_ (mg/L), A_665_ and A_649_ = absorbance at 665 nm and 649 nm, V = extraction volume (10 mL), D = dilution factor, W = fresh weight of sample (g). The factor 1000 converts the extract volume from milliliters to liters because $C_{a}$, $C_{b}$, and $C_{a+b}$ are expressed in mg L^−1^. The numerical coefficients 13.95, 6.88, 24.96, 7.32, 6.63, and 18.08 are assay-specific spectrophotometric coefficients used in the G0601W kit protocol to calculate chlorophyll concentrations from absorbance at 665 and 649 nm.

#### 2.4.2. ROS Accumulation

##### Determination of Superoxide Anion (O_2_^−^)

Superoxide anion (O_2_^−^) content was determined using the hydroxylamine method [21] with a commercial assay kit (G0116W; Geruisi Biotechnology Co., Ltd., Suzhou, China). Approximately 0.10 g of fresh leaf tissue was homogenized on ice with 1 mL of the kit-supplied extraction solution and centrifuged at 12,000 rpm for 10 min at 4 °C. The resulting supernatant was used for analysis. For each reaction, 50 μL of sample extract and 50 μL of reagent 1 were mixed and incubated at 37 °C for 10 min. Subsequently, 100 μL of the reaction mixture supplied with the kit was added, followed by incubation at 37 °C for another 10 min. Absorbance was measured immediately at 540 nm. O_2_^−^ content was calculated using the manufacturer-provided calibration equation:

O_2_^−^ content (nmol g^−1^ FW) = 410 × (ΔA + 0.0011) × D/W

where ΔA represents A_sample_ − A_blank_, D represents the dilution factor, and W represents the fresh weight of the sample (g). The coefficient 410 is the assay-specific calibration and volume-normalization factor used in the G0116W kit to convert the corrected absorbance to O_2_^−^ content, whereas 0.0011 is the intercept-correction term of the kit calibration equation.

##### Determination of Hydrogen Peroxide (H_2_O_2_)

Hydrogen peroxide (H_2_O_2_) content was determined using a titanium-salt colorimetric method [22,23] with a commercial assay kit (G0112W; Geruisi Biotechnology Co., Ltd., Suzhou, China). Approximately 0.10 g of fresh leaf tissue was homogenized on ice with 1 mL of pre-cooled extraction solution and centrifuged at 12,000 rpm for 10 min at 4 °C. For the colorimetric reaction, 500 μL of the supernatant was mixed with 50 μL of reagent 1 and 100 μL of reagent 2. Following centrifugation, the resulting precipitate was retained and dissolved in 460 μL of reagent 3. After clarification, 200 μL of the resulting solution was transferred to a 96-well plate and absorbance was measured at 415 nm. H_2_O_2_ content was calculated from the manufacturer-provided calibration equation (y=2.4544x−0.0329) as follows:

H_2_O_2_ content (µmol g^−1^ FW) = 1.63 × (ΔA + 0.0329) × D/W

where ΔA = A_sample_ − A_blank_, D represents the dilution factor, and W represents the fresh weight of the sample (g). The H_2_O_2_ calibration equation supplied with the assay is y = 2.4544x − 0.0329, where y is ΔA and x is the amount of H_2_O_2_ (µmol). Thus, 0.0329 is the intercept-correction term, whereas 1.63 is the simplified conversion coefficient obtained after incorporating the calibration slope and the fixed extraction and assay volumes.

#### 2.4.3. Antioxidant Enzyme Activities

##### Determination of Superoxide Dismutase (SOD)

Superoxide dismutase (SOD) activity was tested by the nitroblue tetrazolium (NBT) method [24] with a commercial assay kit (G0104F; Geruisi Biotechnology Co., Ltd., Suzhou, China). Approximately 0.10 g of fresh leaf tissue was homogenized on ice with 1 mL of kit extraction buffer and centrifuged at 12,000 rpm for 10 min at 4 °C. The reaction mixture contained 60 μL of sample extract, 240 μL of reagent 1, 60 μL of reagent 2, 60 μL of reagent 3, and 320 μL of reagent 4. After thorough mixing, the reaction was incubated at 25 °C for 30 min and absorbance was measured at 560 nm using a 1 cm glass cuvette. Appropriate blank and sample-background controls were prepared according to the kit protocol. SOD activity was calculated from the inhibition rate (I) as follows:

$$\mathrm{SOD}\;\mathrm{activity}(U\;g^{-1}\;\mathrm{FW})=\frac{12.4\times I}{(1-I)\times W}\times D$$

where I = inhibition rate (as decimal, not %), W = fresh weight of sample (g), D = dilution factor. The coefficient 12.4 is the assay volume-normalization factor derived from the fixed extract volume (V = 1.0 mL), sample volume (V_1_ = 0.06 mL), and total reaction volume (V_2_ = 0.74 mL). One unit (U) of SOD activity was defined according to the kit protocol as the amount of enzyme producing 50% inhibition of NBT reduction under the specified assay conditions.

##### Determination of Peroxidase (POD)

Peroxidase (POD) activity was determined using the guaiacol oxidation method [25] with a commercial assay kit (G0107W; Geruisi Biotechnology Co., Ltd., Suzhou, China). Approximately 0.10 g of fresh leaf tissue was homogenized on ice with 1 mL of extraction buffer and centrifuged at 12,000 rpm for 10 min at 4 °C. The reaction mixture in each well contained 10 μL of sample extract, 40 μL of reagent 1, 140 μL of reagent 2, and 10 μL of reagent 3. Absorbance at 470 nm was measured immediately after mixing ($A_{1}$) and again after 1 min ($A_{2}$); $\Delta A=A_{2}-A_{1}$. POD activity was calculated as follows:

$$\mathrm{POD}\;\mathrm{activity}\;(\Delta {\mathrm{OD}}_{470}\;\min^{-1}\;g^{-1}\;\mathrm{FW})=\frac{100\times \Delta A}{W}\times D$$

where ΔA = change in absorbance at 470 nm per minute, W = fresh weight of sample (g), D = dilution factor. The coefficient 100 is the assay-specific volume- and time-normalization factor specified in the G0107W protocol for converting ΔA_470_ to POD activity. POD activity was expressed as the change in absorbance at 470 nm per minute normalized to 1 g fresh weight.

##### Determination of Catalase (CAT)

Catalase (CAT) activity was determined using the H_2_O_2_ decomposition/colorimetric method [26] with a commercial assay kit (G0106F; Geruisi Biotechnology Co., Ltd., Suzhou, China). Approximately 0.10 g of fresh leaf tissue was homogenized on ice with 1 mL of extraction buffer and centrifuged at 12,000 rpm for 10 min at 4 °C. For the enzymatic reaction, 10 μL of sample extract was mixed with 70 μL of reagent 1 and 20 μL of reagent 2 and incubated at 25 °C for 5 min, after which 100 μL of reagent 3 was added. For color development, 10 μL of this reaction mixture was combined with 900 μL of reagent 1 and 290 μL of reagent 4 and incubated at 25 °C for 5 min. One milliliter was transferred to a 1 cm glass cuvette and absorbance was measured at 510 nm. CAT activity was calculated from $\Delta A=A_{blank}-A_{test}$ using the kit calibration equation (y=0.2093x−0.0025):

$$\mathrm{CAT}\;\mathrm{activity}\;(µ\mathrm{mol}\;\min^{-1}\;g^{-1}\;\mathrm{FW})=\frac{95.6\times \;(\Delta A+0.0025)}{W}\times D$$

where ΔA = A_blank_ − A_test_, W = fresh weight of sample (g), D = dilution factor. The CAT calibration equation is y = 0.2093x − 0.0025, where x is the amount of H_2_O_2_ (µmol) and y is ΔA. Thus, 0.0025 is the intercept-correction term, whereas 95.6 is the simplified conversion coefficient incorporating the calibration slope, extraction volume, sample volume, and reaction time. CAT activity was expressed as µmol H_2_O_2_ decomposed per minute per gram fresh weight.

##### Determination of Ascorbate Peroxidase (APX)

Ascorbate peroxidase (APX) activity was determined using the ascorbate oxidation method [27] with a commercial assay kit (G0203F; Geruisi Biotechnology Co., Ltd., Suzhou, China). Approximately 0.10 g of fresh leaf tissue was homogenized on ice with 1 mL of extraction buffer and centrifuged at 12,000 rpm for 20 min at 4 °C. Reagent 1 was equilibrated at 25 °C for 30 min before measurement. The reaction was performed in a 1 mL quartz cuvette containing 60 μL of sample extract, 740 μL of reagent 1, 100 μL of reagent 2, and 100 μL of reagent 3. After rapid mixing, absorbance at 290 nm was recorded at 30 s (A_1_) and 5 min 30 s (A_2_), and ΔA = A_1_ − A_2_. APX activity was calculated using the molar extinction coefficient of ascorbate at 290 nm (ε = 2.8 × 10^3^ L mol^−1^ cm^−1^):

$$\mathrm{APX}\;\mathrm{activity}\;(µ\mathrm{mol}\;\min^{-1}\;g^{-1}\;\mathrm{FW})\;=\frac{1.19\times \Delta A}{W}$$

where ΔA = change in absorbance at 290 nm per minute, W = fresh weight of sample (g). The coefficient 1.19 is the assay-specific conversion factor derived from the molar extinction coefficient of ascorbate (ε = 2.8 × 10^3^ L mol^−1^ cm^−1^), the 1 cm optical path length, and the fixed extraction and reaction volumes used in the G0203F assay. APX activity was expressed as µmol ascorbate oxidized per minute per gram fresh weight.

#### 2.4.4. Osmotic Adjustment Substances

##### Determination of Proline (PRO)

Proline (PRO) content was determined using the acidic ninhydrin colorimetric method [28] with a commercial assay kit [28] with a commercial assay kit (G0111W; Geruisi Biotechnology Co., Ltd., Suzhou, China). Approximately 0.10 g of fresh leaf tissue was homogenized on ice with 1 mL of the kit-supplied extraction solution. The homogenate was extracted in a 90 °C water bath for 10 min, cooled to room temperature, and centrifuged at 12,000 rpm for 10 min at 25 °C. For the colorimetric reaction, 150 μL of supernatant was mixed with 150 μL of glacial acetic acid and 300 μL of reagent 1 and incubated at 95 °C for 30 min. After cooling to room temperature, 200 μL of the clear reaction mixture was transferred to a 96-well plate and absorbance was measured at 520 nm. Proline content was calculated using the manufacturer-provided standard curve equation (y = 0.1625x − 0.0064):

$$\mathrm{Proline}\;\mathrm{content}\;(µg\;g^{-1}\;\mathrm{FW})\;=\frac{41.03\times (\Delta A+0.0064)}{W}\times D$$

where ΔA = A_sample_ − A_blank_, W = fresh weight of sample (g), D = dilution factor. The proline calibration equation is y = 0.1625x − 0.0064, where x is the amount of proline (µg) and y is ΔA. Thus, 0.0064 is the intercept-correction term and 41.03 is the simplified coefficient obtained from the calibration slope and the fixed extraction and assay volumes.

##### Determination of Soluble Sugar (SS)

Soluble sugar (SS) content was determined using the anthrone sulfuric acid method [29] with a commercial assay kit (G0501W; Geruisi Biotechnology Co., Ltd., Suzhou, China). Approximately 0.10 g of fresh leaf tissue was homogenized on ice with 0.8 mL of 80% (*v*/*v*) ethanol. The homogenate and rinsing solution were combined and brought to a final volume of 1.5 mL with 80% ethanol. Samples were extracted at 50 °C for 20 min, with mixing every 2 min, cooled to room temperature, and centrifuged at 12,000 rpm for 10 min at room temperature. For the reaction, 25 μL of sample extract was mixed with 75 μL of distilled water, 30 μL of anthrone working solution, and 250 μL of concentrated sulfuric acid. The mixture was incubated at 95–100 °C for 10 min and cooled to room temperature. A 200-μL aliquot was transferred to a 96-well plate, and absorbance was measured at 620 nm. Soluble sugar content was calculated using the manufacturer-provided standard curve equation (y = 2.0899x − 0.0103):

$$\mathrm{Soluble}\;\mathrm{sugar}\;\mathrm{content}\;(\mathrm{mg}\;g^{-1}\;\mathrm{FW})\;=\frac{0.718\times (\Delta A+0.0103)}{W}\times D$$

where ΔA = A_sample_ − A_blank_, W = fresh weight of sample (g), D = dilution factor. The glucose calibration equation is y = 2.0899x − 0.0103, where x is the soluble-sugar concentration (mg mL^−1^) and y is ΔA. Thus, 0.0103 is the intercept-correction term and 0.718 is the simplified coefficient incorporating the calibration slope and the fixed extract volume (1.5 mL).

##### Determination of Soluble Protein (SP)

Soluble protein (SP) content was Soluble protein (SP) content was determined using the Biuret method [30] with a commercial assay kit (G0432W; Geruisi Biotechnology Co., Ltd., Suzhou, China). Approximately 0.10 g of fresh leaf tissue was homogenized on ice with 1 mL of extraction buffer and centrifuged at 12,000 rpm for 10 min at 4 °C. The reaction mixture was prepared by combining reagents A and B with ultrapure water at a ratio of 5:3:2 (*v*/*v*/*v*). For analysis, 20 μL of sample extract was mixed with 180 μL of reaction mixture and incubated at 37 °C for 10 min. The entire 200-μL reaction was transferred to a 96-well plate, and absorbance was measured at 540 nm. A 10 mg mL^−1^ protein standard supplied with the kit was used as the reference, and soluble protein content was calculated as:

$$\mathrm{Soluble}\;\mathrm{protein}\;\mathrm{content}\;(\mathrm{mg}\;g^{-1}\;\mathrm{FW})\;=10\;\times \;\Delta A÷(A_{\mathrm{standard}}-A_{\mathrm{blank}})\;÷W\times D$$

where ΔA = A_sample_ − A_blank_, A_standard_ = absorbance of standard, A_blank_ = absorbance of blank, W = fresh weight of sample (g), D = dilution factor. The value 10 corresponds to the concentration of the supplied protein standard (10 mg mL^−1^) used in the G0432W Biuret assay.

#### 2.4.5. Lipid Peroxidation

##### Determination of MDA

The malondialdehyde (MDA) content was determined using the thiobarbituric acid (TBA) assay [31] with a commercial assay kit (G0109W; Geruisi Biotechnology Co., Ltd., Suzhou, China) and the results were expressed as MDA equivalents. Approximately 0.10 g of fresh leaf tissue was homogenized on ice with 1 mL of extraction buffer and centrifuged at 12,000 rpm for 10 min at 4 °C. A 200 μL aliquot of the resulting supernatant was mixed with 300 μL of TBA-containing working solution and incubated at 90–95 °C for 30 min. The samples were subsequently cooled on ice and centrifuged at 12,000 rpm for 10 min at 25 °C. A 200 μL aliquot of the clarified supernatant was transferred to a 96-well microplate, and absorbance was measured at 532 and 600 nm. Non-specific absorbance was corrected as ΔA = A_532_ − A_600_. MDA equivalents were calculated using a molar extinction coefficient of 155 × 10^3^ L mol^−1^ cm^−1^ according to the kit equation:

$$\mathrm{MDA}\;\mathrm{content}\;(\mathrm{nmol}\;g^{-1}\;\mathrm{FW})\;=\;\frac{32.3\times \left(A_{532}-A_{600}\right)}{W}$$

where W represents the fresh weight of the sample. The coefficient 32.3 is the combined conversion factor derived from the MDA molar extinction coefficient (ε = 155 × 10^3^ L mol^−1^ cm^−1^), the optical path length (0.5 cm), the total extraction volume (1.0 mL), the sample volume used in the reaction (0.2 mL), and the final reaction volume (5 × 10^−4^ L), with conversion to nmol g^−1^ FW.

### 2.5. Statistical Analysis

Statistical analyses were performed using GraphPad Prism 8.0, R 4.5.3 software, and Python (statsmodels 0.14.6). Repeated measurements of continuous morphological traits were analyzed using linear mixed-effects models with cultivar, NaCl treatment, time, and their interactions as fixed effects and pot identity as a random effect. The ordinal salt injury index was analyzed separately using an ordinal generalized estimating equation (GEE) model with pot as the clustering unit. Variables measured only at the final sampling time were analyzed by two-way ANOVA with cultivar, NaCl treatment, and their interaction as fixed factors, followed by Tukey’s post hoc test. Day-28 continuous morphological data presented in Figure S2 were analyzed using the same two-way ANOVA and Tukey procedure. Pearson correlation analysis and principal component analysis (PCA) were used as exploratory approaches to examine associations and overall multivariate patterns among measured traits. Statistical significance was accepted at *p* < 0.05. PCA was conducted using individual biological replicate values (*n* = 24), with variables mean-centered and scaled to unit variance before analysis.

## 3. Results

### 3.1. Effects of Salt Stress on Growth and Morphological Parameters of R. rugosa Cultivars

**Figure 1:**
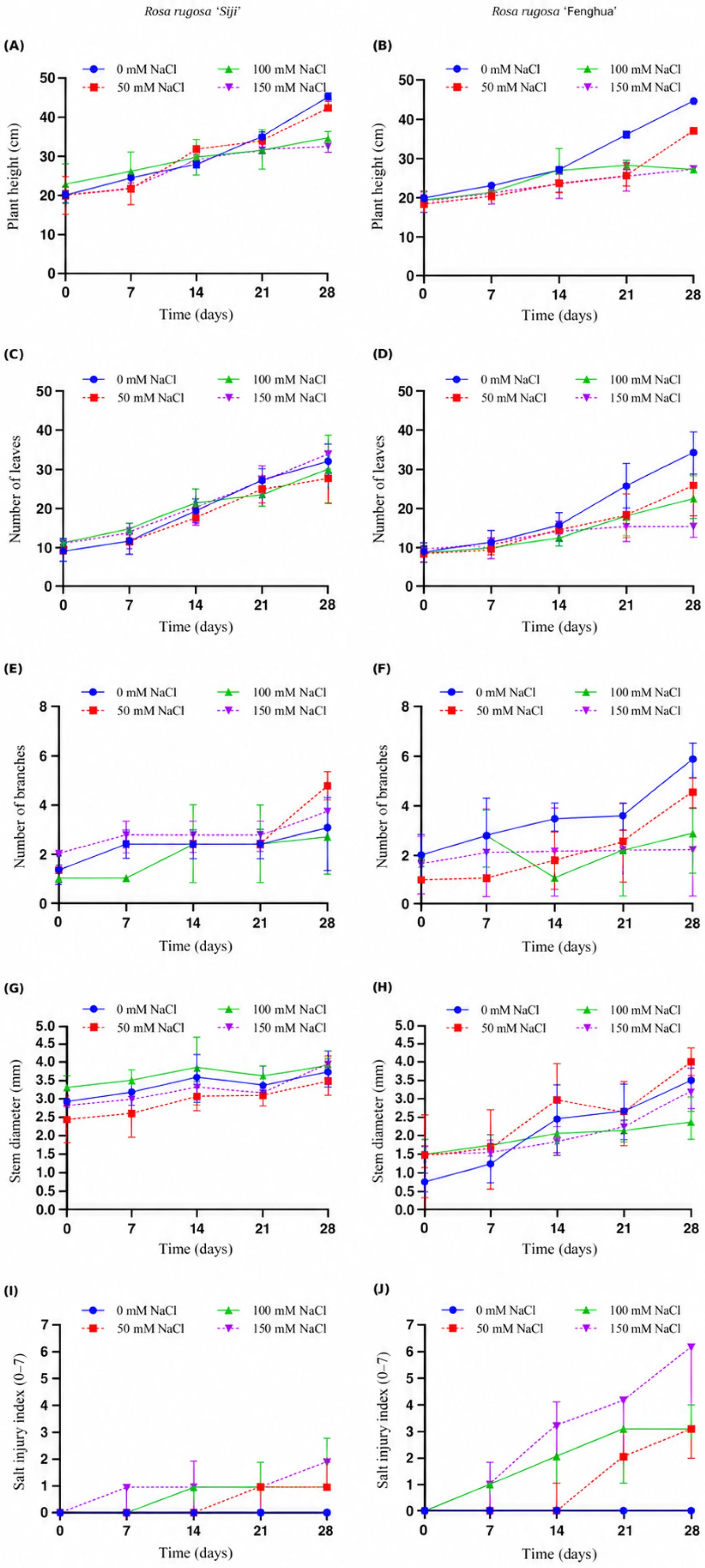
Morphological responses of two *R. rugosa* cultivars to NaCl irrigation treatments. Plant height (**A**,**B**), leaf number (**C**,**D**), branch number (**E**,**F**), stem diameter (**G**,**H**), and salt injury index (**I**,**J**) were recorded at 0, 7, 14, 21, and 28 days. For each trait, the left and right panels represent *Rosa rugosa* ‘Siji’ and *Rosa rugosa* ‘Fenghua’, respectively. Plant height, leaf number, branch number, and stem diameter are presented as mean ± standard deviation (SD) of three biological replicates (*n* = 3), whereas salt injury index values are presented as median with range (*n* = 3). Longitudinal continuous traits were analyzed using linear mixed-effects models testing the effects of cultivar, NaCl treatment, time, and their interactions, with individual pot identity included as a random effect. The ordinal salt injury index was analyzed separately using an ordinal generalized estimating equation (GEE) model with pot as the clustering unit.

Salt stress markedly affected the morphological performance of the two *R. rugosa* cultivars, with the magnitude of response depending on both NaCl concentration and cultivar (Figure 1). Illustrative phenotypic responses further showed progressive visible salt injury with increasing NaCl concentration, including reduced plant vigor, leaf chlorosis, wilting, and growth suppression (Figure S1). Under control conditions, both Siji and Fenghua showed progressive increases in plant height, leaf number, stem diameter, and branch number throughout the 28-day experimental period, indicating normal vegetative growth in the absence of salt stress. However, increasing NaCl concentration gradually suppressed plant growth, particularly at 100 and 150 mM NaCl. Linear mixed-effects analysis revealed a significant effect of time on all four continuous morphological traits (all *p* < 0.001). Significant NaCl treatment × time interactions were also detected for plant height, leaf number, branch number, and stem diameter (all *p* < 0.001), indicating that the effects of NaCl treatment developed over the course of the experiment. Significant cultivar × NaCl treatment × time interactions were observed for plant height, leaf number, and branch number (all *p* < 0.001), whereas this three-way interaction was not significant for stem diameter (*p* = 0.370) (Table S1).

Plant height increased continuously in all treatments, but the rate of increase declined with increasing salinity (Figure 1A,B). Growth suppression became increasingly evident under the higher NaCl treatments as the experiment progressed. In particular, ‘Siji’ maintained comparatively greater plant height than ‘Fenghua’ during the later stages of the 100 and 150 mM treatments. The significant cultivar × NaCl treatment × time interaction (*p* < 0.001) indicates that the temporal response of plant height to NaCl treatment differed between the two cultivars. A similar trend was observed for leaf production (Figure 1C,D). The number of leaves increased over time in both varieties under control and mild salt stress, whereas higher NaCl levels restricted leaf development. This response was particularly apparent in ‘Fenghua’ under 150 mM NaCl during the later stages of treatment, while ‘Siji’ maintained comparatively greater leaf numbers. The significant cultivar × NaCl treatment × time interaction (*p* < 0.001) confirms that the temporal response of leaf production to NaCl treatment differed between the two cultivars. Branch number generally increased during the experimental period, although considerable variation was observed among biological replicates and treatments (Figure 1E,F). The overall main effects of cultivar (*p* = 1.000) and NaCl treatment (*p* = 0.280) were not significant. However, significant NaCl treatment × time and cultivar × NaCl treatment × time interactions were detected (both *p* < 0.001), indicating that changes in branch development over time depended on both NaCl treatment and cultivar. Stem diameter generally increased over time in both cultivars, although the response varied among NaCl treatments (Figure 1G,H). A significant NaCl treatment × time interaction was detected (*p* < 0.001), together with a significant cultivar × NaCl treatment interaction (*p* = 0.009). In contrast, the cultivar × NaCl treatment × time interaction was not significant (*p* = 0.370). These results indicate that stem-diameter responses varied among NaCl treatments over time, although cultivar-specific temporal differences were less pronounced than those observed for plant height, leaf number, and branch number. For clearer visualization of end-point morphological responses, the day-28 values for plant height, number of leaves, number of branches, and stem diameter are additionally presented as grouped bar charts in Figure S2. The salt injury index increased with both treatment duration and NaCl concentration (Figure 1I,J). No visible injury was recorded in control plants, whereas salt-treated plants showed progressively greater injury symptoms over time. The progression of injury was more pronounced in ‘Fenghua’, particularly under the higher NaCl treatments. At day 28, the median salt injury indices under 0, 50, 100, and 150 mM NaCl were 0, 1, 2, and 2, respectively, in ‘Siji’, compared with 0, 3, 3, and 6 in ‘Fenghua’. Ordinal repeated-measures analysis showed significant effects of cultivar, NaCl treatment, and time (all *p* < 0.001), together with significant cultivar × NaCl treatment (*p* = 0.032) and cultivar × NaCl treatment × time (*p* = 0.040) interactions indicating that the temporal progression of salt injury across NaCl treatments differed between the two cultivars.

Taken together, the longitudinal analyses showed that the effects of NaCl treatment on vegetative growth developed progressively over time and differed between the two cultivars for several traits. Under the higher NaCl treatments, ‘Siji’ generally maintained greater plant height and leaf production and developed less severe salt injury than ‘Fenghua’, indicating comparatively better maintenance of vegetative performance under the conditions of this experiment.

### 3.2. Effects of NaCl Stress on Leaf Area and Leaf Fresh and Dry Weight

**Figure 2:**
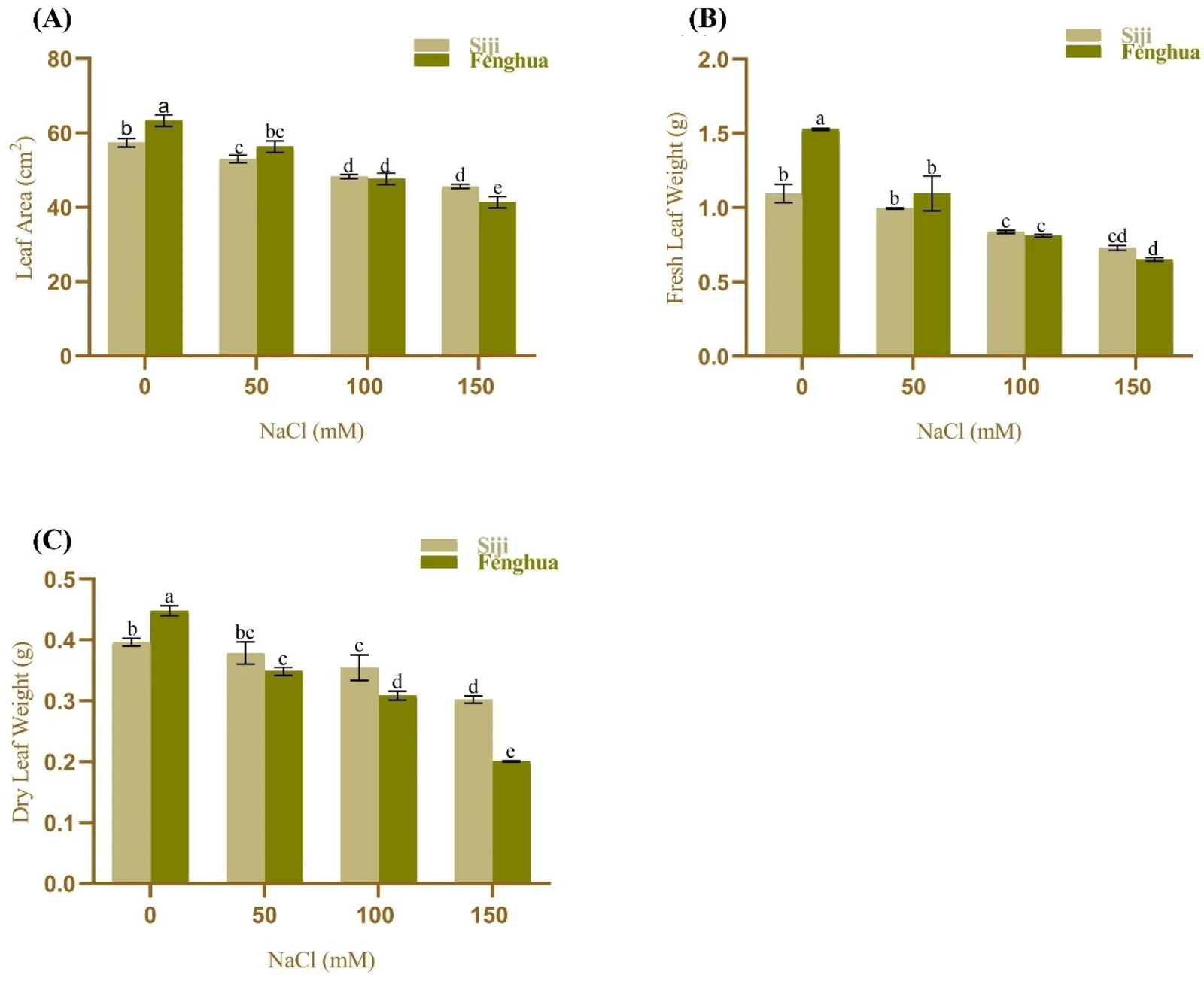
Effects of NaCl treatments on leaf area and leaf fresh and dry weight of *R. rugosa* cultivars. Leaf area (**A**), fresh leaf weight (**B**), and dry leaf weight (**C**) were measured under different NaCl levels (0, 50, 100, and 150 mM). Data are shown as mean ± standard deviation (SD) of three biological replicates (n = 3). Different lowercase letters indicate significant differences among all cultivar × NaCl treatment combinations shown within each panel according to Tukey’s post hoc test following two-way ANOVA (*p* < 0.05).

NaCl treatment significantly affected leaf area and the fresh and dry weight of individual sampled leaves in both *R. rugosa* cultivars, with greater reductions generally observed as NaCl concentration increased (Figure 2). Leaf area, leaf fresh weight/leaf dry weight was highest under non-saline conditions and gradually declined with increasing NaCl concentration. These measurements represent individual leaf-level responses and should not be interpreted as estimates of whole-plant biomass.

Leaf area presented a clear decreasing pattern in response to increasing NaCl levels (Figure 2A). Under control conditions, Fenghua exhibited a significantly greater leaf area than Siji. However, leaf area decreased markedly in both cultivars under salt stress. At 50 mM NaCl, both cultivars maintained relatively higher leaf area compared with the severe stress treatments, whereas exposure to 100 and 150 mM NaCl caused further reductions. At the highest salinity level, Siji maintained a slightly larger leaf area than Fenghua, suggesting that Siji was better able to sustain leaf expansion under severe salt stress. Fresh leaf weight was also significantly influenced by NaCl treatment (Figure 2B). The highest fresh leaf weight was noted in Fenghua under control conditions, followed by Siji. Increasing NaCl treatment resulted in a gradual decline in fresh leaf weight in both cultivars, with the reduction becoming more pronounced at 100 and 150 mM NaCl. the higher NaCl treatments, ‘Fenghua’ showed a greater reduction in fresh leaf weight than ‘Siji’, consistent with the stronger reduction in mass observed in this cultivar. Dry leaf weight tailed a similar pattern to fresh leaf weight (Figure 2C). Control plants showed the highest dry leaf weight, while NaCl-treated plants exhibited a concentration-dependent decline. Fenghua had the greatest dry leaf weight under control conditions, but its dry biomass decreased sharply as salinity increased. In contrast, Siji maintained comparatively higher dry leaf weight under 100 and 150 mM NaCl. At 150 mM NaCl, Fenghua showed the lowest dry leaf weight, whereas Siji retained a greater proportion of dry biomass, suggesting better tolerance to salinity-induced growth inhibition.

These results show that NaCl stress reduced individual leaf area and leaf fresh and dry weight in both cultivars. Although ‘Fenghua’ showed higher values for several leaf-level traits under control conditions, ‘Siji’ retained comparatively higher leaf area and leaf dry weight under 100 and 150 mM NaCl. Because whole-plant biomass was not measured, these results are interpreted specifically as leaf-level responses.

### 3.3. Effects of NaCl Stress on ROS Accumulation

**Figure 3:**
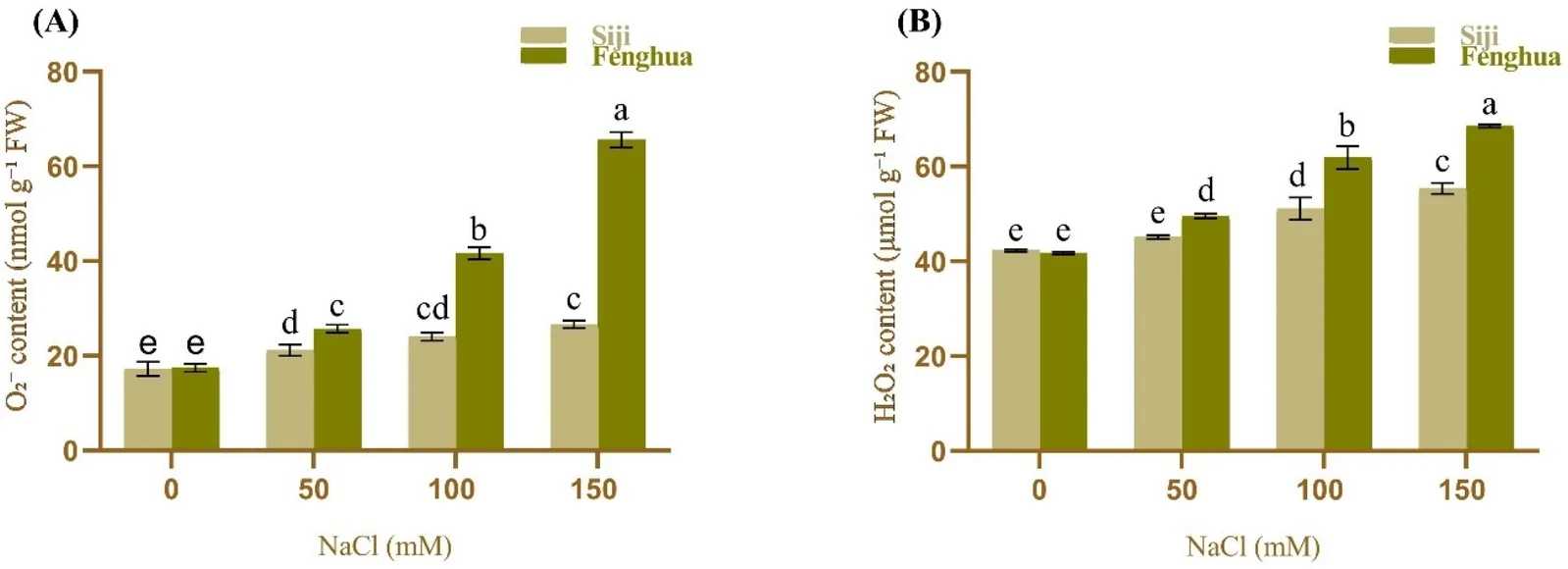
Effects of NaCl irrigation treatments on reactive oxygen species (ROS) accumulation in *R. rugosa* cultivars. Superoxide anion (O_2_^−^) content (**A**) and hydrogen peroxide (H_2_O_2_) content (**B**) were determined under different NaCl concentrations (0, 50, 100, and 150 mM). Data are presented as mean ± standard deviation (SD) of three biological replicates (n = 3). Different lowercase letters indicate significant differences among all cultivar × NaCl treatment combinations shown within each panel according to Tukey’s post hoc test following two-way ANOVA (*p* < 0.05).

NaCl treatment led to a pronounced rise in reactive oxygen species (ROS) accumulation in both *R. rugosa* cultivars, as indicated by alterations in superoxide anion (O_2_^−^) and hydrogen peroxide (H_2_O_2_) levels (Figure 3). Under control conditions, O_2_^−^ content remained relatively low and showed only minor differences between Siji and Fenghua. However, increasing NaCl concentration progressively enhanced O_2_^−^ accumulation in both cultivars. This increase was more pronounced in Fenghua, particularly under moderate and severe salt stress. At 100 and 150 mM NaCl, Fenghua showed markedly higher O_2_^−^ content than Siji, indicating stronger salt-induced oxidative pressure in this variety. In contrast, Siji maintained comparatively lower O_2_^−^ accumulation under the same NaCl treatments.

A comparable pattern was also detected for hydrogen peroxide (H_2_O_2_) content (Figure 3B). H_2_O_2_ levels increased progressively with increasing NaCl concentration in both varieties, but the magnitude of increase differed between them. Fenghua accumulated substantially higher H_2_O_2_ under 100 and 150 mM NaCl, whereas Siji showed a more moderate increase. The lower accumulation of O_2_^−^ and H_2_O_2_ in Siji under severe salinity suggests that this variety experienced less ROS accumulation than Fenghua.

Thus, sustained NaCl stress promoted ROS accumulation in a concentration-dependent manner. The higher O_2_^−^ and H_2_O_2_ levels in Fenghua, particularly at 100 and 150 mM NaCl, indicate a stronger oxidative response than in Siji.

### 3.4. Effects of NaCl Stress on Antioxidant Defense Enzyme Activities

**Figure 4:**
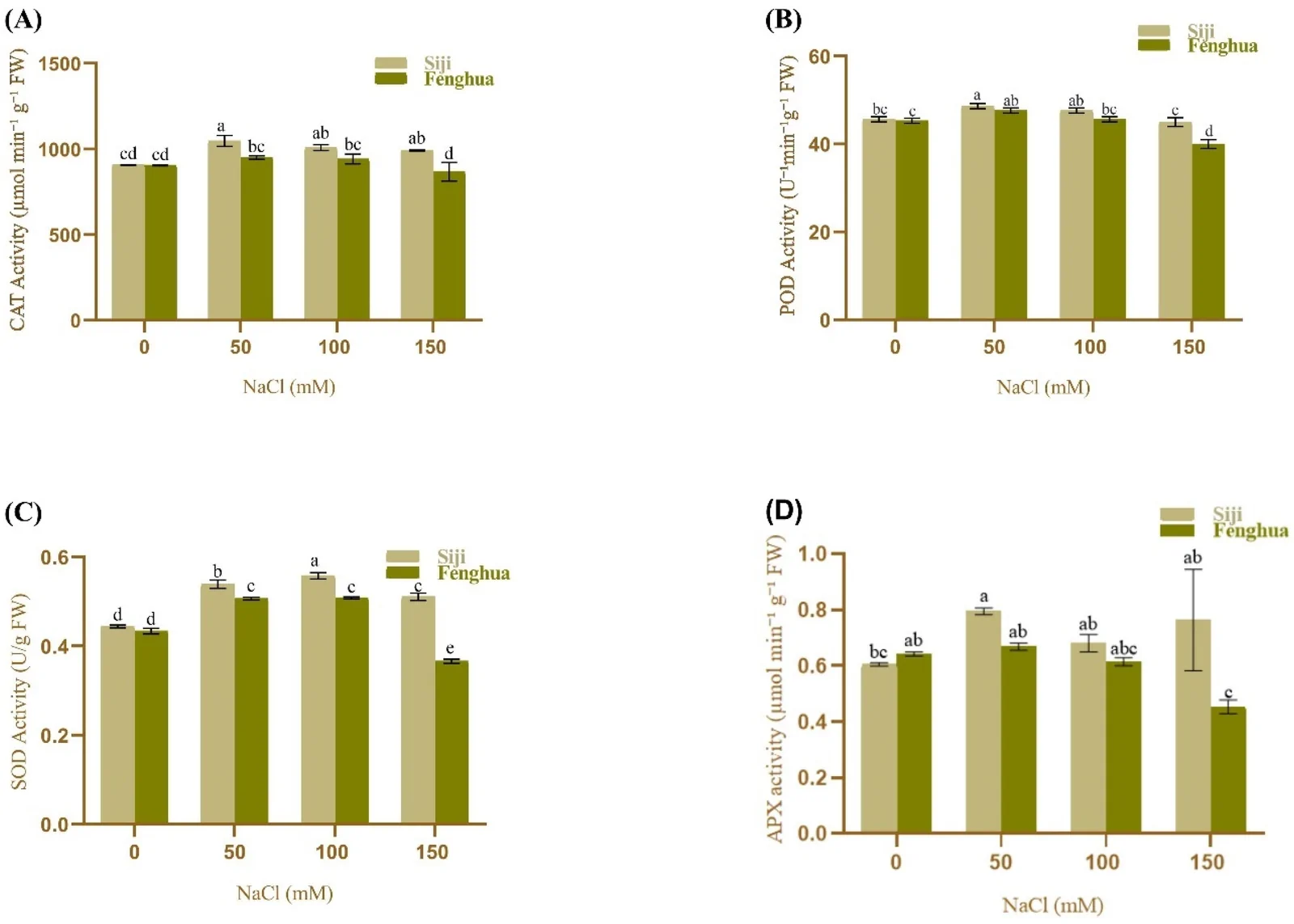
Effects of NaCl treatments on antioxidant enzyme activities in *R. rugosa* cultivars. The activities of catalase (CAT) (**A**), peroxidase (POD) (**B**), superoxide dismutase (SOD) (**C**), and ascorbate peroxidase (APX) (**D**) were evaluated under different NaCl concentrations. Data are presented as mean ± standard deviation (SD) of three biological replicates (n = 3). Different lowercase letters indicate significant differences among all cultivar × NaCl treatment combinations shown within each panel according to Tukey’s post hoc test following two-way ANOVA (*p* < 0.05).

Following the increase in ROS accumulation, NaCl stress markedly altered antioxidant enzyme activities in the two *R. rugosa* cultivars, indicating activation of enzymatic defense mechanisms under salinity (Figure 4). The activities of catalase (CAT), peroxidase (POD), superoxide dismutase (SOD), and ascorbate peroxidase (APX) varied significantly among NaCl treatments and between cultivars, suggesting genotype-dependent differences in antioxidant regulation under salt stress.

CAT activity increased under salt stress compared with the control, particularly in Siji (Figure 4A). In Siji, CAT activity reached its maximum at 50 mM NaCl and remained relatively high at 100 and 150 mM NaCl. In contrast, Fenghua showed only a slight increase at 50 and 100 mM NaCl, followed by a clear reduction at 150 mM NaCl. The comparatively higher CAT activity maintained by ‘Siji’ under the higher NaCl treatments coincided with its lower H_2_O_2_ accumulation, indicating stronger maintenance of CAT activity under these conditions. POD activity also responded positively to salt stress, especially under mild and moderate NaCl treatments (Figure 4B). Both cultivars showed increased POD activity at 50 mM NaCl compared with the control. However, Siji consistently maintained higher POD activity than Fenghua across salt treatments. At 150 mM NaCl, POD activity declined in both cultivars, but the reduction was more pronounced in Fenghua. This indicates that Siji retained a more stable antioxidant response under severe salinity, whereas Fenghua showed reduced enzymatic protection at the highest NaCl concentration. SOD activity showed a distinct stress-induced increase, particularly in Siji (Figure 4C). Under control conditions, both cultivars exhibited similar SOD activity. With increasing NaCl concentration, SOD activity increased significantly, reaching the highest level in Siji at 100 mM NaCl. Although Siji showed a slight reduction at 150 mM NaCl, its SOD activity remained higher than that of Fenghua. In Fenghua, SOD activity increased under 50 and 100 mM NaCl but declined sharply at 150 mM NaCl. The comparatively stronger SOD activity in Siji suggests more efficient conversion of superoxide radicals into hydrogen peroxide, representing an important first-line defense against salt-induced reactive oxygen species. APX activity displayed a variety-specific response to NaCl stress (Figure 4D). In Siji, APX activity increased under salt treatment and remained relatively high even at 150 mM NaCl, although the variation was greater at the highest concentration. Fenghua showed a slight increase under mild stress but declined markedly at 150 mM NaCl. The reduction in APX activity in ‘Fenghua’ at 150 mM NaCl contrasted with the comparatively greater maintenance of APX activity in ‘Siji’, further demonstrating cultivar-specific differences in antioxidant enzyme responses.

Overall, Siji maintained higher CAT, POD, SOD, and APX activities than Fenghua under most salt-stress treatments, whereas Fenghua showed a marked decline in several enzyme activities at 150 mM NaCl. This pattern indicates a stronger maintenance of enzymatic antioxidant capacity in Siji under severe salinity.

### 3.5. Effects of NaCl Stress on Osmolyte Accumulation, Photosynthetic Pigments, and Lipid Peroxidation

**Figure 5:**
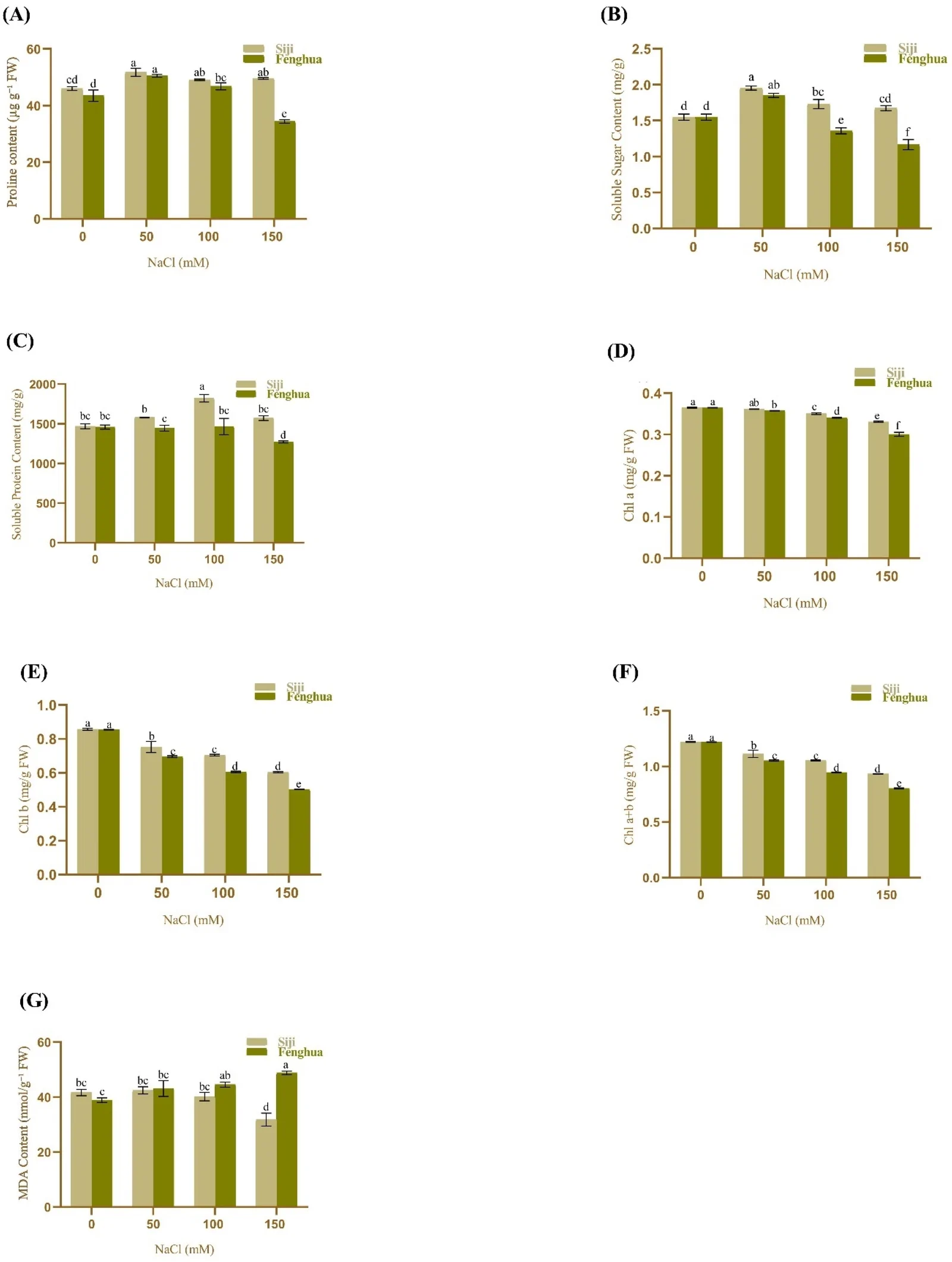
Impact of NaCl treatments on biochemical responses in *R. rugosa* cultivars. Osmolytes, including proline (PRO) (**A**), soluble sugars (SS) (**B**), and soluble proteins (SP) (**C**); photosynthetic pigments, including chlorophyll a (Chl a) (**D**), chlorophyll b (Chl b) (**E**), and total chlorophyll (Chl a + b) (**F**); and lipid peroxidation, represented by malondialdehyde (MDA) content (**G**), were assessed under different NaCl concentrations. Data are presented as mean ± standard deviation (SD) of three biological replicates (n = 3). Different lowercase letters indicate significant differences among all cultivar × NaCl treatment combinations shown within each panel according to Tukey’s post hoc test following two-way ANOVA (*p* < 0.05).

NaCl treatment altered osmolyte accumulation, photosynthetic pigment contents, and lipid peroxidation in both *R. rugosa* cultivars, although the magnitude and pattern of these responses differed between ‘Siji’ and ‘Fenghua’ (Figure 5; Table S2).

Proline (PRO), soluble sugars (SS), and soluble proteins (SP) showed non-linear and cultivar-specific responses to NaCl treatment rather than a uniform concentration-dependent increase (Figure 5A–C). In ‘Siji’, PRO increased from 46.0 µg g^−1^ FW in the control to 51.7 µg g^−1^ FW at 50 mM NaCl and remained comparatively high at 100 and 150 mM NaCl (49.1 and 49.6 µg g^−1^ FW, respectively). SS also increased under mild NaCl treatment, reaching its maximum at 50 mM NaCl, but subsequently declined at 100 and 150 mM. SP showed a different response, reaching its highest level at 100 mM NaCl before decreasing at 150 mM. These responses indicate that osmolyte adjustment in ‘Siji’ was most pronounced at mild to moderate NaCl treatments rather than increasing continuously with treatment concentration.

In ‘Fenghua’, PRO and SS similarly increased under the mild NaCl treatment but declined as the treatment concentration increased further. The reduction was particularly pronounced at 150 mM NaCl, where PRO and SS decreased to 34.4 µg g^−1^ FW and 1.165 mg g^−1^ FW, respectively. SP also declined markedly at the highest NaCl treatment. Thus, although both cultivars showed osmolyte responses under NaCl treatment, ‘Siji’ generally maintained comparatively higher levels of these compounds than ‘Fenghua’ under the higher treatments. This pattern is consistent with better maintenance of osmotic-adjustment-related metabolites in ‘Siji’ under the conditions of the experiment.

In contrast to the non-linear osmolyte responses, chlorophyll pigments generally declined with increasing NaCl treatment in both cultivars (Figure 5D–F). Chlorophyll a, chlorophyll b, and total chlorophyll were reduced most clearly under the higher NaCl treatments, indicating progressive loss of photosynthetic pigments under salinity. The decline was more pronounced in ‘Fenghua’, whereas ‘Siji’ retained comparatively higher pigment contents, particularly at 150 mM NaCl; total chlorophyll at this treatment was 0.935 mg g^−1^ FW in ‘Siji’ compared with 0.803 mg g^−1^ FW in ‘Fenghua’. These results indicate greater chlorophyll retention in ‘Siji’ under severe NaCl treatment, consistent with lower salt-induced pigment loss.

MDA showed a contrasting cultivar-specific response to NaCl treatment (Figure 5G). In ‘Siji’, MDA remained relatively similar to the control under 50 and 100 mM NaCl but decreased significantly at 150 mM NaCl, from 41.63 nmol g^−1^ FW in the control to 31.78 nmol g^−1^ FW. In contrast, MDA increased in ‘Fenghua’ with NaCl treatment, reaching 48.79 nmol g^−1^ FW at 150 mM NaCl. Therefore, the highest NaCl treatment was associated with substantially lower MDA accumulation in ‘Siji’ than in ‘Fenghua’, indicating a contrasting cultivar-specific pattern of membrane lipid peroxidation. The lower MDA level in ‘Siji’ under severe NaCl treatment was consistent with its comparatively lower oxidative damage under the tested conditions.

Taken together, the biochemical responses to NaCl treatment were strongly cultivar-dependent. ‘Siji’ showed better maintenance of several osmolytes under moderate to severe treatment, greater chlorophyll retention, and lower MDA accumulation at the highest NaCl treatment, whereas ‘Fenghua’ showed marked declines in osmolytes and chlorophyll pigments together with increased MDA accumulation under severe treatment. These contrasting responses were associated with the better overall performance of ‘Siji’ under the conditions of this experiment.

### 3.6. Correlation Analysis Among Morpho-Physiological Traits

**Figure 6:**
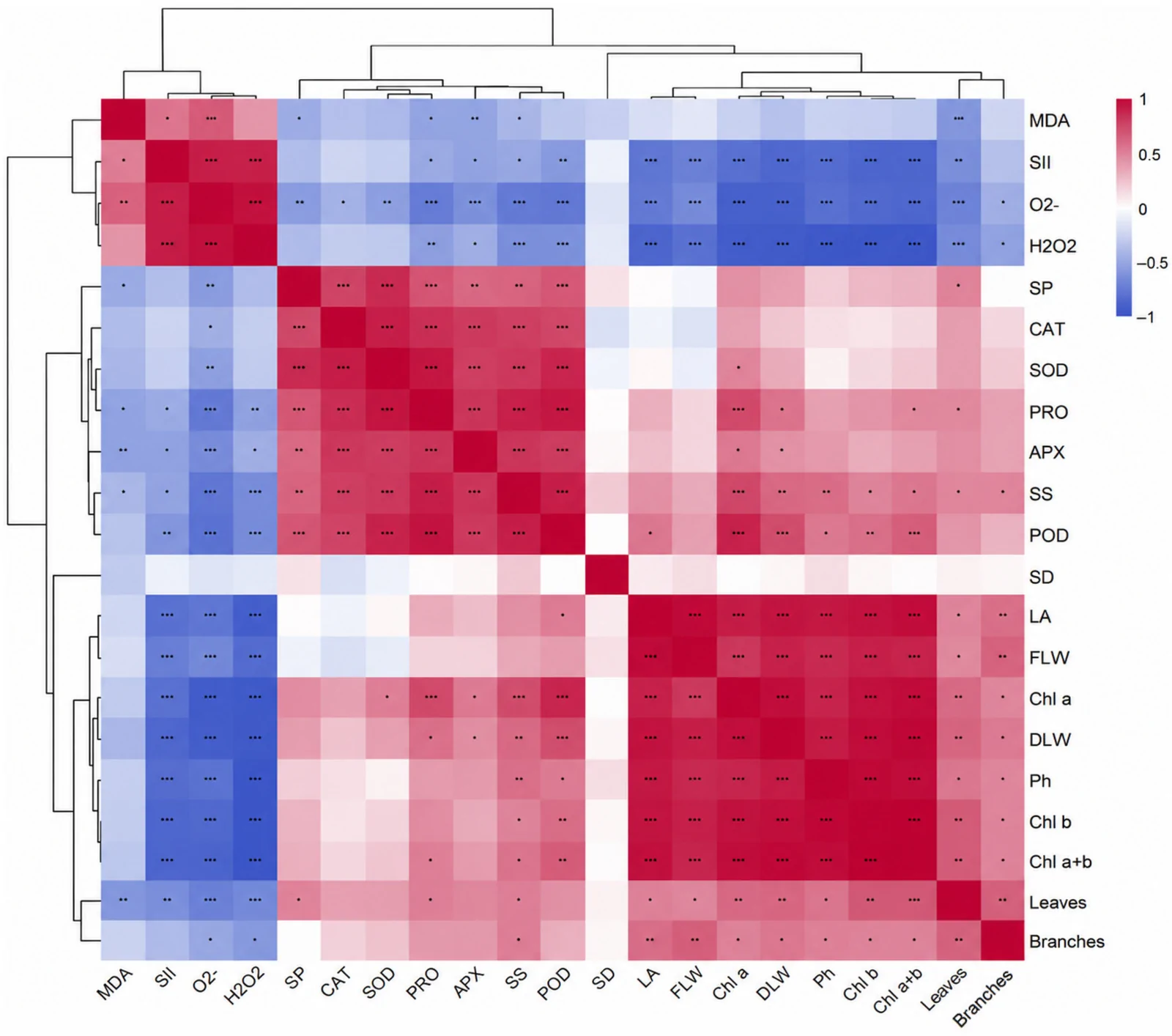
Pearson correlation coefficients among physiological and biochemical traits of *R. rugosa* under different NaCl treatments. Significant correlations are shown at *p* < 0.05 (*), *p* < 0.01 (**) and *p* < 0.001 (***). Abbreviations: Ph, plant height; SD, stem diameter; Leaves, number of leaves; Branches, number of branches; SII, salt injury index; LA, leaf area; FLW, fresh leaf weight; DLW, dry leaf weight; O_2_^−^, superoxide anion; H_2_O_2_, hydrogen peroxide; SOD, superoxide dismutase; POD, peroxidase; CAT, catalase; APX, ascorbate peroxidase; PRO, proline; SS, soluble sugars; SP, soluble proteins; Chl a, chlorophyll a; Chl b, chlorophyll b; Chl a + b, total chlorophyll; MDA, malondialdehyde.

Pearson correlation analysis was undertaken to assess the relationships among antioxidant enzyme activities, osmolyte accumulation, photosynthetic pigments, and lipid peroxidation in *R. rugosa* under NaCl stress (Figure 6). The results revealed strong positive correlations among antioxidant enzymes, indicating a coordinated enzymatic response under salinity. SOD, POD, CAT, and APX were positively and significantly correlated with one another, suggesting that these enzymes responded in parallel to NaCl-induced stress.

Osmolyte-related traits also showed close associations with antioxidant enzyme activities. Proline and soluble sugars showed positive correlations with all antioxidant enzymes, whereas soluble protein exhibited positive correlations with most antioxidant enzymes. These relationships indicate a close association between osmolyte-related traits and antioxidant enzyme activities under NaCl treatment. Photosynthetic pigments showed positive correlations with several antioxidant and osmolyte-related traits. Chlorophyll a was positively correlated with SOD, POD, APX, proline, and soluble sugars, whereas chlorophyll b and total chlorophyll were positively related mainly with POD and soluble sugars. Strong positive correlations were also detected among chlorophyll a, chlorophyll b, and total chlorophyll, confirming consistency among photosynthetic pigment traits. In contrast, MDA showed negative correlations with most antioxidant enzymes, osmolytes, and chlorophyll traits. Significant negative correlations were observed between MDA and APX, proline, soluble sugars, and soluble protein, indicating that higher antioxidant activity and osmolyte accumulation were generally associated with lower lipid peroxidation. ROS-related traits showed clear associations with oxidative damage and growth inhibition. O_2_^−^ and H_2_O_2_ were positively associated with stress injury-related traits, including MDA and salt injury index, indicating that increased ROS accumulation was closely linked with membrane lipid peroxidation and visible salt injury under NaCl stress. In contrast, O_2_^−^ and H_2_O_2_ showed negative associations with several growth, biomass, and chlorophyll-related traits, suggesting that higher ROS accumulation was accompanied by reduced growth performance and photosynthetic pigment retention. These relationships indicate that ROS accumulation, antioxidant enzyme activities, osmolyte-related traits, and membrane lipid peroxidation were closely associated during the responses to NaCl treatment in *R. rugosa*. These correlations are exploratory and describe patterns of association among the measured traits; they do not establish direct causal relationships.

### 3.7. Principal Component Analysis of Morpho-Physiological and ROS-Related Traits

**Figure 7:**
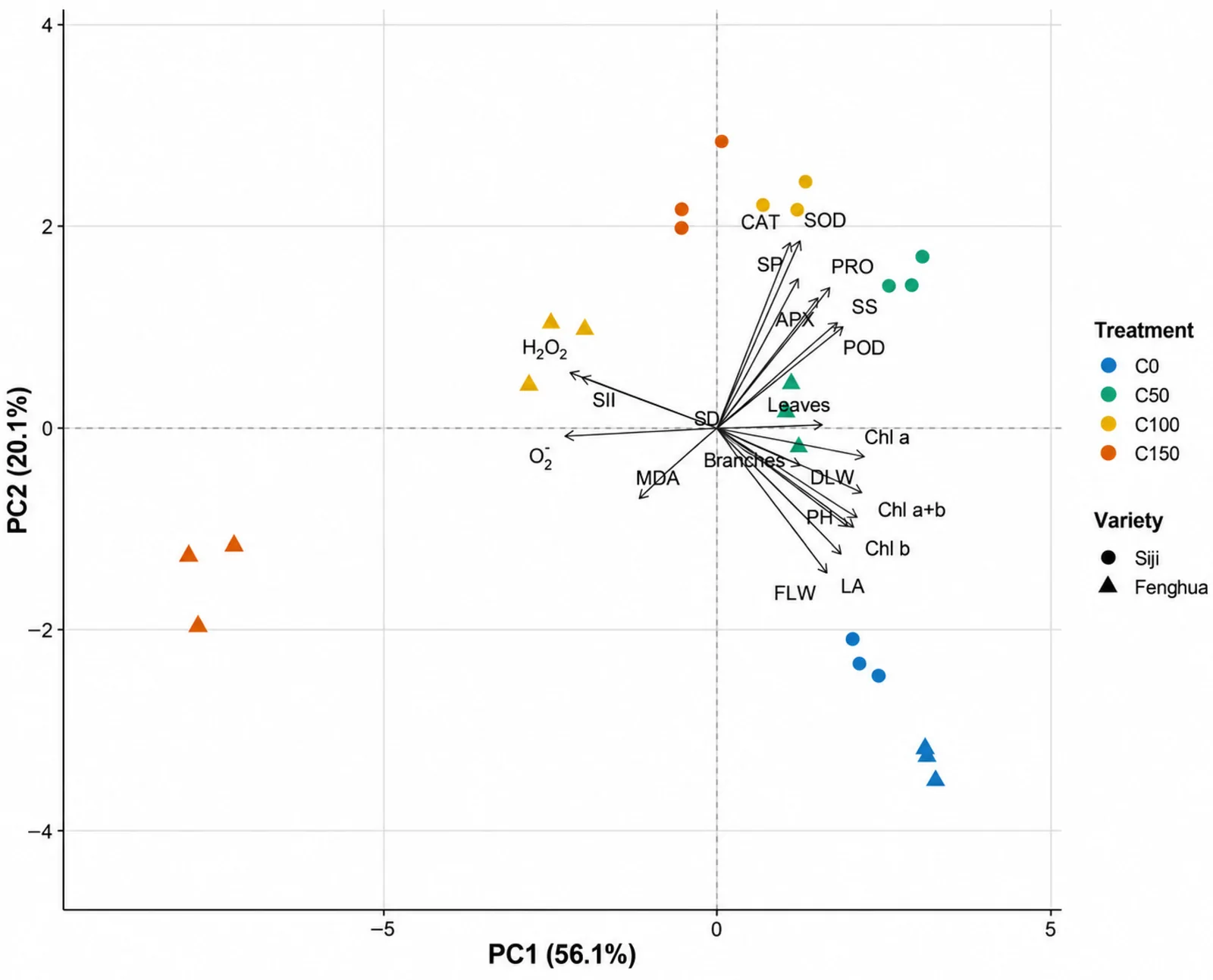
Principal component analysis (PCA) biplot showing the relationships among morpho-physiological, biochemical, and ROS-related traits of *R. rugosa* cultivars under different NaCl treatments. PC1 and PC2 explained 56.1% and 20.1% of the total variation, respectively. PCA was performed using individual biological replicate values (*n* = 24), with all variables mean-centered and scaled to unit variance before analysis. Points represent individual samples, with colors indicating NaCl treatments and shapes representing varieties. Arrows indicate the contribution and direction of each trait. Abbreviations are as defined in Figure 6.

Principal component analysis (PCA) was used to explore multivariate associations among morphological, physiological, biochemical, and ROS-related traits and to visualize response patterns of *Rosa rugosa* cultivars under different NaCl treatments (Figure 7). The analysis was based on 24 individual biological replicates using centered and scaled variables. PC1 and PC2 explained 56.1% and 20.1% of the total standardized variation, respectively.

The PCA biplot showed clear separation among NaCl treatments, suggesting that salt stress substantially altered the physiological and biochemical profiles of *R. rugosa*. Control treatments were mainly positioned close to growth, leaf-level mass, and chlorophyll-related traits, including plant height, leaf area, fresh leaf weight, dry leaf weight, chlorophyll a, chlorophyll b, and total chlorophyll. This indicates that plants under non-saline conditions maintained better vegetative growth, leaf-level mass accumulation, and photosynthetic pigment status. In contrast, salt-stressed treatments were separated from the controls and were more closely associated with stress-response traits. The arrows for O_2_^−^, H_2_O_2_, MDA, and salt injury index were positioned toward the left side of the biplot, indicating their association with oxidative stress, lipid peroxidation, and visible salt injury. Fenghua samples under higher NaCl concentrations were positioned closer to these stress-related traits, especially under 100 and 150 mM NaCl, suggesting stronger ROS accumulation and oxidative damage in this variety. Antioxidant enzyme and osmolyte-related traits, including SOD, CAT, POD, APX, PRO, SS, and SP, were mainly located in the upper-right region of the biplot. Siji samples under salt stress were positioned closer to these protective traits than Fenghua, indicating stronger antioxidant defense and osmotic adjustment responses. This separation suggests that Siji maintained a more favorable physiological and biochemical profile under salinity, whereas Fenghua was more strongly associated with ROS accumulation, membrane damage, and salt injury.

The PCA therefore separated control and salt-stressed treatments mainly along gradients of growth/chlorophyll traits and oxidative-stress-related traits. Siji samples under salinity were positioned closer to antioxidant and osmolyte traits, whereas Fenghua samples under higher NaCl levels were more closely associated with ROS accumulation, MDA, and salt injury index. Accordingly, these PCA patterns are interpreted as exploratory multivariate associations and not as evidence of causal or mechanistic relationships among traits.

## 4. Discussion

The present study demonstrates that sustained NaCl treatment altered vegetative growth, ROS accumulation, chlorophyll content, osmolyte profiles, antioxidant enzyme activities, and membrane lipid peroxidation in *R. rugosa*, with the magnitude and pattern of these responses differing between the two cultivars. Under the higher NaCl treatments, ‘Fenghua’ showed greater salt injury, higher O_2_^−^ and H_2_O_2_ accumulation, greater MDA accumulation, and stronger reductions in chlorophyll and leaf-level mass, whereas ‘Siji’ generally maintained lower ROS accumulation, stronger antioxidant enzyme activities, better maintenance of several osmolytes, and greater chlorophyll and retention of leaf area and leaf fresh/dry weight. Taken together, these patterns indicate that the comparatively better performance of ‘Siji’ under NaCl treatment was associated with coordinated differences in redox status, osmolyte maintenance, and membrane lipid peroxidation rather than with a uniform response of any single physiological trait.

The reduction in morphological and leaf-level traits under salinity indicates that NaCl stress restricted vegetative growth of *R. rugosa*. Such growth suppression is generally attributed to osmotic limitation, ion toxicity, and reduced cellular expansion under salinity [18]. The stronger retention of growth and leaf area and leaf fresh/dry weight in ‘Siji’ indicates comparatively better maintenance of vegetative performance under NaCl treatment. This finding agrees with recent studies on *R. rugosa* and other plant species showing that salt-tolerant genotypes generally maintain higher biomass and lower injury symptoms than sensitive genotypes under saline conditions [32]. Qi et al. reported that *R. rugosa* exhibits active morphological, physiological, and transcriptomic responses to salt stress, supporting the idea that growth-related traits are closely linked with salinity adaptation in this species [18]. Similarly, Han et al. described *R. rugosa* as a suitable model for studying saline–alkali tolerance because of its natural occurrence in coastal environments and its ability to activate multiple physiological and molecular defense pathways under stress [33]. The present study extends these findings by directly comparing two *R. rugosa* cultivars and showing that varietal differences in salt tolerance are clearly reflected in growth preservation and reduced salt injury.

Salt stress also caused a reduction in chlorophyll content, indicating that NaCl negatively affected photosynthetic pigment stability in *R. rugosa*. Chlorophyll degradation under salinity may result from ionic toxicity, oxidative damage to chloroplast membranes, inhibition of chlorophyll biosynthesis, and accelerated pigment breakdown. Recent studies have reported that salinity reduces chlorophyll content by enhancing ROS production, damaging photosynthetic proteins, and disturbing photosystem organization [34]. Similarly, studies on salt-stressed plants have shown that treatments or genotypes with improved tolerance often maintain higher chlorophyll content, better photosynthetic efficiency, and stronger antioxidant activity under 100–150 mM NaCl stress [35]. In the present study, however, chlorophyll concentrations were measured; therefore, the comparatively greater chlorophyll retention in ‘Siji’ should be interpreted as improved pigment retention rather than direct evidence of maintained photosynthetic capacity, photosynthetic efficiency, or protection of chloroplast function. These findings are consistent with the redox-based model of salt injury, in which excessive ROS production disrupts chloroplast function, impairs photosynthetic electron transport, accelerates chlorophyll degradation, and promotes membrane lipid peroxidation [5,36,37]. Similar ROS-associated responses have also been reported in rose-related salt-stress studies, where stronger ROS detoxification and antioxidant regulation were linked with reduced oxidative injury and improved stress adaptation [6,18]. These ROS patterns were associated with a stronger antioxidant response, greater chlorophyll retention, and lower membrane damage observed in ‘Siji’ under the higher NaCl treatments.

The cultivar-specific differences in O_2_^−^ and H_2_O_2_ accumulation coincided with differences in antioxidant enzyme activities. SOD participates in the conversion of O_2_^−^ to H_2_O_2_, whereas CAT, POD, and APX contribute to H_2_O_2_ metabolism. Therefore, the comparatively stronger maintenance of these enzyme activities in ‘Siji’ is consistent with its lower ROS accumulation under the higher NaCl treatments; however, the present data do not establish a direct causal relationship between enzyme activities and ROS levels. This interpretation is consistent with salinity studies showing that tolerant genotypes commonly maintain stronger antioxidant enzyme activities and lower oxidative damage than sensitive genotypes [12]. Han et al. reported that saline–alkali stress in *R. rugosa* stimulated physiological and transcriptomic responses involving oxidative stress regulation, secondary metabolism, and photosynthesis-related pathways [33]. Another recent study on *R. rugosa* found that salt stress affected SOD and POD activities and altered soluble sugar and MDA contents in roots and leaves, confirming that antioxidant defense and osmotic regulation are central to salt response in this species [38]. Recent studies have emphasized that osmolyte accumulation helps plants maintain cellular hydration and metabolic activity under salt stress and often works together with antioxidant defense pathways [39].

The non-linear osmolyte responses observed in the present study indicate that osmotic adjustment was not characterized by a uniform increase in compatible solutes with increasing NaCl treatment. Instead, ‘Siji’ generally maintained several osmolyte-related traits more effectively than ‘Fenghua’ under the higher NaCl treatments, whereas marked declines were evident in ‘Fenghua’ under severe salinity. Proline and soluble sugars are known to contribute to osmotic balance and to the stabilization of proteins and cellular membranes during salt stress [40]. Therefore, the comparatively greater maintenance of these compounds in ‘Siji’ is consistent with a more sustained osmotic-adjustment response rather than greater osmolyte accumulation per se. In addition, the positive associations between osmolyte-related traits and antioxidant enzyme activities in the present study suggest that these responses occurred in parallel. Similar coordination between osmolyte status and antioxidant responses has been reported in salt-stressed plants [41], although the correlations observed here do not establish a direct causal relationship. In parallel, the contrasting MDA response was particularly evident under severe NaCl treatment. In ‘Siji’, MDA did not increase progressively with NaCl treatment and was significantly lower at 150 mM NaCl than under control conditions, whereas ‘Fenghua’ showed increased MDA accumulation at the highest NaCl treatment. MDA is widely used as an indicator of oxidative membrane damage under abiotic stress because it reflects membrane lipid peroxidation associated with excessive ROS. Thus, the lower MDA level observed in ‘Siji’ under severe NaCl treatment is consistent with comparatively lower membrane lipid peroxidation. This response coincided with lower O_2_^−^ and H_2_O_2_ accumulation and comparatively stronger maintenance of antioxidant enzyme activities in ‘Siji’, suggesting that more effective control of oxidative stress may have contributed to limiting lipid peroxidation. However, because membrane lipid composition and MDA metabolism were not measured, the mechanism underlying the decrease in MDA cannot be established directly from the present study. This contrasting response agrees with previous research showing that improved salt tolerance is often associated with enhanced antioxidant capacity and lower MDA accumulation. For example, a study on RrLBD40 reported that overexpression of this gene in *R. rugosa* increased POD activity and reduced MDA content under salt stress, indicating an association between enhanced antioxidant defense and reduced lipid peroxidation [42]. Similar results have been reported in other salt-stressed plants, where tolerant genotypes or stress-alleviating treatments maintained lower MDA levels by increasing antioxidant enzyme activities and osmolyte accumulation [39]. Together, these findings support the view that membrane protection is an important component of salt-stress adaptation in *R. rugosa*. The correlation and PCA analyses further indicate that the contrasting salt responses were associated with a coordinated pattern involving ROS accumulation, antioxidant enzyme activities, osmolyte-related traits, chlorophyll retention, and membrane lipid peroxidation.

The findings of this study contribute to the understanding of cultivar-specific physiological and biochemical responses to salinity in *R. rugosa*. Although *R. rugosa* is recognized as a salt-tolerant coastal species, comparative physiological and biochemical information on salinity responses among its cultivars remains limited. Recent transcriptomic and physiological studies have identified several salt-responsive pathways in *R. rugosa*, including antioxidant defense, osmotic regulation, photosynthesis, ion homeostasis, cell wall modification, and secondary metabolism [33]. The present study adds to this knowledge by showing that the contrasting responses of the two cultivars to NaCl treatment were associated with measurable physiological and biochemical traits. In particular, the combination of stronger maintenance of antioxidant enzyme activities and several osmolytes, greater chlorophyll and retention of leaf area and leaf fresh/dry weight, lower ROS accumulation, and reduced MDA accumulation under severe NaCl treatment provides a clear physiological framework for identifying salt-tolerant *R. rugosa* germplasm. From a practical perspective, the tolerant cultivar identified in this study may be useful for cultivation in salt-affected soils, coastal landscapes, ecological restoration programs, and breeding programs aimed at improving stress tolerance in ornamental roses.

## 5. Limitations and Future Perspectives

The present study provides an integrated comparison of morphological, physiological, and biochemical responses of two *R. rugosa* cultivars under NaCl stress; however, several aspects warrant further investigation. First, the experiment was conducted under controlled pot conditions using defined NaCl irrigation concentrations. Although this approach enabled consistent comparison between cultivars, future studies incorporating continuous monitoring of root-zone salinity dynamics and evaluation under more complex environmental conditions would further improve understanding of cultivar performance under practical saline environments. Second, this study focused on physiological and biochemical indicators associated with salt tolerance, including ROS accumulation, antioxidant enzyme activities, osmolyte responses, and lipid peroxidation. These measurements provide valuable evidence of cultivar differences but do not fully resolve the molecular regulatory mechanisms underlying salt adaptation. Future investigations integrating transcriptomic, metabolomic, and redox-related analyses, including glutathione metabolism, would provide deeper insight into the regulatory networks contributing to salinity tolerance in *R. rugosa*. Third, chlorophyll content was evaluated as an indicator of pigment stability under NaCl stress, whereas direct measurements of photosynthetic performance were not included. Future studies combining chlorophyll fluorescence, gas-exchange measurements, and other functional physiological approaches would help determine how differences in pigment retention contribute to photosynthetic performance under salinity. Overall, these future directions will complement the present findings and contribute to a more comprehensive understanding of the mechanisms underlying cultivar-dependent salt tolerance in *R. rugosa*.

## 6. Conclusions

The present study revealed clear cultivar-specific differences in the response of *R. rugosa* to sustained NaCl treatment, with ‘Siji’ exhibiting greater tolerance than ‘Fenghua’ under the controlled experimental conditions. The coordinated maintenance of antioxidant enzyme activities, osmolyte status, chlorophyll pigments, and leaf area, and leaf fresh and dry weight, together with lower ROS accumulation and reduced lipid peroxidation under severe treatment, was consistently associated with the superior performance of ‘Siji’. These findings highlight the importance of integrated redox regulation, osmotic adjustment, pigment retention, and membrane stability in distinguishing contrasting salt-stress responses between *R. rugosa* cultivars. Accordingly, ‘Siji’ represents a promising candidate for further evaluation under saline substrate and field conditions and may provide useful physiological traits for future salt-tolerance screening and breeding in ornamental roses.

## Data Availability

Due to privacy and ethical restrictions, the data supporting the findings of this study are available from the corresponding authors upon reasonable request. Summary data are provided in the article and its Supplementary Materials.

## Supplementary Materials

The following supporting information can be downloaded at: https://www.mdpi.com/article/10.3390/antiox15091218/s1, Figure S1: Illustrative phenotypic responses of *Rosa rugosa* cultivars ‘Siji’ and ‘Fenghua’ to NaCl irrigation treatment; Figure S2: Day-28 morphological responses of *Rosa rugosa* cultivars ‘Siji’ and ‘Fenghua’ to NaCl irrigation treatments; Table S1: Statistical analysis of longitudinal morphological traits and salt injury index; Table S2: Day-28 descriptive statistics of morphological, physiological, biochemical, and oxidative-stress-related traits of *Rosa rugosa* cultivars under NaCl irrigation treatments; Table S3: Pearson correlation coefficients among morphological, physiological, biochemical, and oxidative-stress-related traits of *Rosa rugosa* under NaCl irrigation treatments.
